# Molecular Karyotyping in Children and Adolescents with Gender Dysphoria

**DOI:** 10.1089/trgh.2017.0051

**Published:** 2018-08-01

**Authors:** Ken C. Pang, Debi Feldman, Ralph Oertel, Michelle Telfer

**Affiliations:** ^1^ Department of Adolescent Medicine, Royal Children's Hospital, Parkville, Australia.; ^2^ Murdoch Children's Research Institute, Parkville, Australia.; ^3^ Department of Paediatrics, University of Melbourne, Parkville, Australia.; ^4^ Department of Psychiatry, University of Melbourne, Parkville, Australia.; ^5^ The Walter and Eliza Hall Institute of Medical Research, Parkville, Australia.; ^6^ Victorian Clinical Genetics Service, Parkville, Australia.

**Keywords:** adolescents, children, gender dysphoria, genetics, karyotyping

## Abstract

**Purpose:** The presence of a disorder of sexual development (DSD) acts as a diagnostic specifier for gender dysphoria (GD) under DSM-5, while the International Classification of Diseases (ICD)-10 specifically states that its equivalent diagnosis, gender identity disorder (GID), must not be the result of a chromosomal abnormality. For these reasons, routine karyotyping has been previously advocated in the clinical work-up of children and adolescents with suspected GD or GID. However, the utility of such testing remains unclear.

**Methods:** The results of routine molecular karyotyping were analyzed in 128 patients attending our Australian statewide pediatric gender service from 2013 to 2016. Karyotyping was performed using an Illumina BeadChip platform and provided information on both sex chromosome composition and copy number variation (CNV).

**Results:** No sex chromosome abnormalities directly suggestive of a DSD were discovered. The rate of CNVs among our patient cohort was 8.6% (11/128), similar to that previously reported for the general population. Unexpectedly, three trans male patients shared the same CNV, involving an almost identical 400 kbp deletion on chromosome 15q11.2. The frequency of this deletion within birth-assigned females in our cohort (3/69; 4.3%) was significantly higher than that within local control populations (0.3%; Fisher's exact test *p*-value=0.002), suggesting a possible association between 15q11.2 deletions and trans male identity.

**Conclusion:** Routine molecular karyotyping failed to detect any occult DSD and indicated that the rate of CNVs was similar to that of the general population. Given these findings, we suggest that molecular karyotyping has minimal clinical utility in the routine management of children and adolescents with GD.

## Introduction

Gender dysphoria (GD) describes when an individual experiences incongruence between their sex assigned at birth and their inner gender identity.^1^ In contrast, disorders of sexual development (DSDs) refer to a range of congenital conditions involving anomalies of the chromosomes, gonads, and/or genitalia.^2,3^ Although GD and DSDs are distinct entities, they can co-occur,^4^ especially in DSDs where genetically XY individuals have defects in testosterone synthesis^5^ and genetically XX individuals have excessive androgens due to congenital adrenal hyperplasia.^6^

The presence of a DSD has been an exclusion criterion for the diagnosis of GD in previous versions of the DSM, and is a diagnostic specifier for GD in the current version of DSM-5.^1^ Although some have advocated for the removal of this DSD specifier, its inclusion reflects not only that individuals with GD and a DSD are clinically distinct from those with GD alone but also that treatment criteria differ.^7,8^

Thus, when assessing an individual with suspected GD for the first time, it is important to know whether he/she also has a DSD. In the vast majority of cases where GD and a DSD coexist, this is relatively straightforward since the diagnosis of a DSD has already been made.^8^ However, some types of DSDs—for example, complete androgen insensitivity syndrome (CAIS) and XY gonadal dysgenesis in birth-assigned females (BAFs), and Klinefelter syndrome (KS) in birth-assigned males (BAMs)—typically do not cause clinical symptoms until adolescence and may be undiagnosed. In such cases, the diagnosis of GD may therefore precede suspicion of a DSD, especially in individuals whose gender identity concerns manifest early in life.

How then can such individuals with an occult DSD be identified when assessing for possible GD? In BAFs, a history of amenorrhea may be suggestive of either CAIS or XY gonadal dysgenesis (the former may also be associated with previous inguinal hernia), while physical examination may reveal reduced/absent pubic or axillary hair in both these conditions. In postpubertal BAMs, a history of infertility, gynecomastia, delayed puberty, as well as speech and learning difficulties may be indicative of KS, while physical examination may reveal increased height, reduced body hair and small testicular size in such individuals. However, in prepubertal children, history and examination are likely to be unrewarding, unless there is a history of inguinal hernia or speech and learning difficulties. One potential means to identify individuals with an occult DSD is karyotyping, which evaluates chromosomal composition. For example, in BAFs with occult CAIS or XY gonadal dysgenesis, karyotyping will signal the presence of an underlying DSD by revealing XY instead of XX chromosomes, while in BAMs with occult KS, karyotyping will reveal XXY instead of XY chromosomes.

Performing karyotyping in the context of a medical assessment for GD may be of potential value for other reasons. Molecular karyotyping, which uses genetic technology known as a chromosomal microarray, provides detailed genomic information at a much higher resolution than cytogenetic “G banding” techniques used in conventional karyotyping. Having superseded conventional karyotyping in clinical care in recent years,^9^ molecular karyotyping allows for the identification of genetic anomalies known as copy number variations (CNVs), which can include small chromosomal deletions, duplications, and rearrangements. Although there is no empirical evidence to suggest that there is an increased incidence of CNVs in individuals with GD, it is now well recognized that GD commonly coexists with Autism Spectrum Disorder (ASD),^10–14^ a neurodevelopmental condition that results in impaired social communication and behavior^1^ and is known to be associated with an increased risk of pathogenic CNVs.^15^ Given the latter, the American Academy of Pediatrics now recommends that molecular karyotyping be offered to all patients with ASD.^16^ Whether such testing might also be clinically useful for children and young people with GD—∼50% of whom score in the clinical range for ASD, using the Social Responsiveness Scale screening tool^12,13^—is unclear.

In addition to the DSM, the World Health Organization's International Classification of Diseases (ICD) is the other standard tool for the diagnosis of GD. In the most recent version (ICD-10), gender identity disorder (GID)—as GD is referred to—has three criteria, one of which specifically states that GID must not be the symptom of a chromosomal abnormality.^17^ Thus, it could be argued that karyotyping should be routinely performed in the diagnostic workup of individuals with GD (or GID), and indeed this is commonly what is done in many countries even though it is not explicitly part of current international clinical guidelines.^8,18,19^

Despite this, empirical data on the utility of karyotyping in the management of GD are limited. Between 2003 and 2013, five separate studies of transgender adults using conventional G banding techniques were reported (Table 1).^18,20–23^ Taken together, these studies indicate an overall rate of chromosomal abnormalities in 4/286 trans males (1.4%) and 11/481 trans females (2.3%), both of which are higher than the reported 0.5% prevalence of abnormalities within the general population using conventional karyotyping techniques.^24^ Of the observed abnormalities, six involved sex chromosome abnormalities. Of these, four individuals had KS (three of which had already been diagnosed previously), one had mosaic Turner syndrome (with no pubertal or hormonal disturbance), and the remaining was mosaic 47XYY/46XY. Given the low prevalence of chromosomal abnormalities in transgender individuals observed in these studies, the prevailing recommendation was that the routine use of conventional karyotyping was of limited clinical utility and that karyotyping should be reserved for cases where suspicions of a DSD are raised based on other clinical features.

**Table 1.** **Summary of Previous Karyotyping Studies in Transgender Adults**

	Chromosome abnormalities		
Study	Trans males (BAF), *n* (%)	Trans females (BAM), *n* (%)	Sex chromosome abnormalities
Hengstschlager et al.^22^	0/31 (0)	1/30 (3.3)	Nil detected
Wylie and Steward^20^	0/6 (0)	1/46 (2.2)	One BAM with 46XY/47,XYY (mosaic)
Vujovic et al.^21^	0/76 (0)	0/71 (0)	Nil detected
Inoubli et al.^18^	1/117 (0.9)	8/251 (3.2)	Three BAMs with KS (two already diagnosed)
Auer et al.^23^	3/56 (5.4)	1/83 (1.2)	One BAM with KS (already diagnosed)
			One BAF with Turner's syndrome (mosaic)
Above studies combined	4/286 (1.4)	11/481 (2.3)	6/767 (0.8)

The differences in prevalence between BAF and BAM in each of the individual studies (and when combined) were not statistically significant (Fisher exact test, *p*>0.05).

BAF, birth-assigned female; BAM, birth-assigned male; KS, Klinefelter syndrome.

However, such recommendations do not necessarily apply to a pediatric population, in whom—for reasons already noted above—clinical evidence of some DSDs may not yet be evident. Indeed, on this basis, it has been advocated that the routine use of karyotyping to screen for DSDs should be performed in pediatric cases of suspected GD.^23^ Nevertheless, the clinical utility of karyotype testing in a pediatric population with suspected GD is currently unknown. Moreover, since previously reported studies in adult transgender populations have all used conventional G banding techniques to assess karyotype, it is also unclear whether chromosomal microarrays—which provide ≥10-fold improvement in resolution^9^—might provide additional clinically useful information, especially in light of the increased rates of ASD among those with GD.

The Royal Children's Hospital Gender Service (RCHGS) services a population of over 5 million people in the state of Victoria, Australia. From 2013, young people presenting to the service with suspected GD have routinely been offered molecular karyotyping to analyze chromosomal composition and evaluate for the presence of an occult DSD. Given the current gaps in knowledge, the aim of this study was therefore to determine the clinical utility of molecular karyotyping in a pediatric population with suspected GD.

## Patients and Methods

### Patients

All children and adolescents presenting to the RCHGS from 2013 to 2016 with suspected GD were assessed by a mental health practitioner (either a psychologist or psychiatrist). In those cases where puberty was likely to be imminent or had already commenced, individuals were also seen by a pediatrician within the service. As part of the pediatric assessment, patients routinely received a physical examination (including Tanner staging) as well as hormonal measurement (including luteinizing hormone [LH], follicle-stimulating hormone [FSH], testosterone, and estrogen) and were offered molecular karyotyping to analyze chromosomal composition and evaluate for the presence of an occult DSD.

### Molecular karyotyping

Molecular karyotyping was performed on blood samples at the Victorian Clinical Genetics Service (VCGS) using Illumina CytoSNP or CoreExome BeadChip platforms, which interrogate between 300,000 and 500,000 single-nucleotide polymorphisms across the genome. Molecular karyotyping is the default method of karyotyping at VCGS and was provided at no cost to the patients.

### Data collection and statistical analysis

Molecular karyotyping results were assessed via retrospective audit by cross-referencing known patients of the RCHGS between 2013 and 2016 with VCGS molecular karyotyping records. To compare the prevalence of 15q11.2 deletions within our patient population and that of a local control group, VCGS molecular karyotyping records were also extracted from 4869 abortus material samples that were obtained from either fetal tissue or placental tissue of fetal origin and referred to VCGS for molecular karyotyping due to spontaneous miscarriage or abnormal fetal morphology. Chi-square with Yates correction and Fisher's exact test were used to calculate two-tailed *p*-values to determine whether the 15q11.2 deletion was overrepresented within our patient cohort compared to published and local control groups, respectively. To determine whether the average ages of BAFs and BAMs were significantly different, a two-tailed *p*-value was calculated using Student's *t*-test. In each case, an α of 0.05 was used as a cutoff for significance. Ethics approval for conducting this audit was granted by the Royal Children's Hospital Human Research Ethics Committee.

## Results

### Sample characteristics

One hundred and twenty-eight molecular karyotypes were extracted from 69 BAFs and 59 BAMs. BAFs were significantly older (mean age: 14.7±3.3 years) than the BAMs (mean age: 13.6±3.5 years; Student's *t*-test, *p*<0.05) at the time of testing. Out of 128, 112 individuals were subsequently diagnosed with GD based on DSM-5 criteria by their treating clinicians. For those 16 cases not diagnosed with GD, some failed to attend sufficient appointments to have the diagnosis confirmed, some were continuing to explore and/or were unsure of their gender identity, while others did not have GD.

### Karyotype analysis

In 117/128 (92.1%) of our patients, the molecular karyotype was normal. This meant that the rate of CNVs observed within our patient cohort was 8.6% (11/128). Of the 11 abnormal karyotypes detected (Table 2), 5 were from BAM and 6 from BAF, and no sex chromosome abnormalities directly suggestive of a DSD were identified. However, a 530 kbp duplication within the X chromosome (Xq26.3) was detected in one BAM with a female gender identity and a 46XY karyotype, although the clinical significance of this was reported as “unknown.” Of the remaining ten autosomal CNVs, eight deletions and two duplications were identified.

**Table 2.** **Molecular Karyotype Changes and Clinical Features**

Patient	Birth assigned gender	Gender identity	Age (years)^a^	Additional clinical features	Molecular karyotype^b^		CNV description	CNV classification	CNV-associated clinical features	References
1	BAM	Female^c^	8	GDD, ADHD, suspected ASD, ODD, facial dysmorphism	46XY	22q11.21(18,801,657-21,460,220)x3	22q11.21 Duplication	Pathogenic	GDD, ADHD, ASD, ODD, facial dysmorphism	^25^
2	BAM	Female	13	Nil	46XY	22q11.21(20,708,641-21,460,220)x1	22q11.21 deletion	Uncertain significance	ID, facial dysmorphism, behavioral problems, urogenital anomalies	^46^
3	BAF	Male	18	Nil	46XX	15q11.2(22,770,994-23,236,972)x1	15q11.2 Deletion	Uncertain significance	Speech delay, motor delay, behavioral problems, ASD, seizures	^26,39^
4	BAF	Male	17	ASD	46XX	15q11.2(22,754,322-23,222,284)x1	15q11.2 Deletion	Uncertain significance	As above	
5	BAF	Male	6	ID, ASD, ADHD	46XX	15q11.2(22,750,305-23,135,794)x1	15q11.2 Deletion	Uncertain significance	As above	
6	BAM	Female^c^	8	Nil	46XY	17q11.2 (28,967,865-29,324,994)x1	17q11.2 Deletion	Uncertain significance	Overgrowth, learning difficulties, facial dysmorphism	^47^
7	BAM	Female	12	Nil	46XY	3q25.32(158,369,915-158,778,092)x1	3q25.32 Deletion	Unknown significance	Nil	
8	BAF	Male	15	Nil	46XX	9p24.3(1,022,010-1,142,653)x1	9p24.3 deletion	Unknown significance	Nil	
9	BAF	Male	11	ASD, ADHD	46XX	15q26.3(99,550,237-100,093,949)x3	15q26.3 Duplication	Unknown significance	Nil	
10	BAF	Male	16	Nil	46XX	16q23.3(83,825,658-84,181,023)x1	16q23.3 deletion	Unknown significance	Nil	
11	BAM	Female	17	Nil	46XY	Xq26.3(133,810,888-134,339,576)x2	Xq26.3 Duplication	Unknown significance	Nil	

^a^ At time of molecular karyotype testing.

^b^ Chromsomal coordinates correspond to hg19 genome assembly.

^c^ Originally referred after expressing desire to be female, but currently identifying as mostly male.

ADHD, attention-deficit hyperactivity disorder; ASD, Autism Spectrum Disorder; CNV, copy number variation; GDD, global developmental delay; ID, intellectual disability; ODD, oppositional defiant disorder.

Only one of these was classified as a pathogenic change, and involved a 2.6 Mbp duplication on chromosome 22 (22q11.21). Similar duplications have been associated with a variety of clinical features, including global developmental delay, ASD, attention-deficit hyperactivity disorder (ADHD), oppositional defiant disorder (ODD), and dysmorphic facial features, with variable expressivity.^25^ Consistent with this, our particular patient had previously been diagnosed with global developmental delay, ADHD, and ODD, was suspected of having ASD, and was subsequently noted to have facial features in keeping with those described in others with a 22q11.21 duplication.

Four other CNVs observed within our patients were reported as being of “uncertain significance.” In each of these cases, the CNV had been previously associated with a range of clinical phenotypes that displayed incomplete penetrance (i.e., some individuals with the CNV were apparently normal). One of these CNVs was located on chromosome 22q11.21—similar to that of our other patient—but this one involved a deletion rather than a duplication. Unexpectedly, three of the other CNVs of “uncertain significance”—all of which were present in BAFs with a male gender identity—involved almost identical 400 kbp deletions on chromosome 15q11.2. In previous studies, this deletion has been found to occur at an overall rate of 0.3% in healthy control populations (264/100,466).^26^ Notably, the rate observed within not only our BAFs (3/69; 4.3%) but also our entire patient cohort (3/128; 2.3%) was significantly higher than within these controls (Chi-square with Yates correction, *p*<0.0001). However, since the incidence of 15q11.2 deletions can vary between different populations, we also compared our observed rates with that of a local control group. This group consisted of abortus material samples referred to VCGS for molecular karyotyping due to spontaneous miscarriage or abnormal fetal morphology, and 16/4869 (0.3%) had 15q11.2 deletions, which was again significantly different to the rate observed among our patients (Fisher's exact test, *p*=0.002 for BAFs and *p*=0.01 for the entire cohort). Taken together, these data raise the possibility that 15q11.2 deletions might confer susceptibility to GD and, more specifically, trans male identity.

The remaining four CNVs were not previously associated with any clinical phenotype and were therefore reported as being of “unknown significance.”

## Discussion

The number of children and adolescents being referred for specialist medical care with suspected GD has increased dramatically in recent years.^27–32^ With population-based surveys now estimating that 1.2% of teenagers identify as transgender,^33^ it is quite likely that referral rates will continue to grow. Knowing how best to assess and treat children and adolescents with suspected GD is therefore of increasing relevance to pediatric practice. In this regard, previous authors have suggested that karyotyping to screen for DSDs should be routinely performed in pediatric cases of suspected GD,^23^ but the clinical utility of doing so remains unknown.

In this study, we present for the first time molecular karyotyping results for a large cohort of children and adolescents presenting with suspected GD. Our routine molecular karyotyping of 128 children and adolescents failed to detect any occult DSD. Although one BAM displayed structural variation at a chromosomal locus (Xq26.3) that has been previously implicated in XX sex reversal,^34^ our patient was genetically 46XY and showed no clinical evidence of a DSD, suggesting that the duplication is simply an incidental finding. Taken together, our results are therefore consistent with earlier studies of transgender adults,^18,20–23^ which suggested that routine karyotyping was of limited clinical benefit in identifying occult DSDs.

Unlike previous karyotyping studies in transgender adults, our study used molecular karyotyping methods that provide much higher resolution than conventional G-banding techniques and can thus detect small chromosomal changes that are otherwise missed with conventional karyotyping.^9^ Although the precision of such methods carries potential disadvantages (e.g., detection of coincidental genetic changes whose significance may be uncertain), our use of molecular karyotyping was potentially important for two main reasons, both of which related to a desire to explore the genetic basis of GD, support for which comes from previous twin studies.^35–37^

First, as noted earlier, individuals with GD show much higher rates of ASD than expected by chance,^10^ suggesting that there might be a shared developmental vulnerability underlying the two conditions. With this in mind—and given that ASD is known to be associated with an increased risk of pathogenic CNVs^15^—we hypothesized that molecular karyotyping might similarly reveal an increased rate of pathogenic CNVs in our patients. However, only 1/128 karyotypes (0.8%) was reported as containing a pathogenic CNV, which is equivalent to the rate expected within the general population.^38^ And, even though the discovery of this particular CNV was clinically useful (e.g., by providing the family with a genetic basis for some of their child's existing difficulties and by facilitating reproductive counseling), the individual involved had multiple clinical features that were suggestive of an underlying genetic cause (e.g., global developmental delay, possible ASD, facial dysmorphism) and should have been an independent prompt for molecular karyotyping. Thus, we would argue that our results do not support a role for routine molecular karyotyping as a screen for pathogenic CNVs in suspected GD, but that molecular karyotyping be offered in a targeted manner to patients with comorbid and unexplained intellectual disability, global developmental delay, and/or ASD, consistent with existing clinical recommendations for a general pediatric population.^16^

Second, high-resolution molecular karyotyping offered an opportunity to explore whether any particular CNVs—regardless of whether or not they were reported as pathogenic—might be associated with GD. Interestingly, even though the overall rate of CNVs within our patient cohort (8.6%) was no higher than that previously observed within the general population,^38^ we unexpectedly found almost identical deletions at chromosome 15q11.2 in three trans male patients. Deletion of this region disrupts four genes, each of which are highly expressed in the brain, and confers susceptibility to a range of neurodevelopmental disorders, including developmental delay, ASD, schizophrenia, and epilepsy.^26,39^ Given that the frequency of 15q11.2 deletions within our patient cohort was significantly higher than that of controls, our findings provide for the first time evidence that this deletion might increase susceptibility to a trans male identity and, more generally, highlight the potential advantages of adopting an unbiased genome-wide approach to understanding gender identity. After all, for over a decade, multiple groups have attempted to explore the genetic basis of transgender identity by looking at variants within specific candidate genes (e.g., estrogen receptor β, androgen receptor, CYP17), but no consistent associations have been found.^40–45^

Finally, it is important to acknowledge the limitations of our study, which principally center on the relatively low number of molecular karyotypes that were assessed. Specifically, having only analyzed 128 molecular karyotypes, we cannot exclude the possibility that occult DSDs might be usefully detected at very low frequency with this technology. Nevertheless, many such DSDs will result in an abnormal hormonal profile during puberty, so endocrine evaluation is likely to provide an alternative and less expensive screening option in this regard. Our relatively low numbers similarly limited the power of our study to detect specific CNVs associated with GD and, in this regard, our finding of a significant association between a trans male identity and 15q11.2 deletion was quite unexpected. However, given our association signal was derived from just 3/69 BAFs, it will be critical to see whether this apparent association can be confirmed in future research studies using larger patient cohorts.

## Conclusion

Routine molecular karyotyping of 128 children and adolescents presenting with suspected GD failed to detect any occult DSD and indicated that the rate of CNVs was similar to that of the general population. We therefore suggest that molecular karyotyping has minimal clinical utility in the routine management of GD.

## Abbreviations Used

ADHD: attention-deficit hyperactivity disorder
ASD: Autism Spectrum Disorder
BAF: birth-assigned female
CAIS: complete androgen insensitivity syndrome
CNV: copy number variation
DSD: disorder of sexual development
GD: gender dysphoria
GID: gender identity disorder
ICD: International Classification of Diseases
KS: Klinefelter syndrome
ODD: oppositional defiant disorder
RCHGS: Royal Children's Hospital Gender Service
VCGS: Victorian Clinical Genetics Service
